# Effectiveness of nutrition interventions targeting university-level student populations across the League of Arab States: a systematic scoping review

**DOI:** 10.1177/17579759241270957

**Published:** 2024-09-30

**Authors:** Elizabeth Dodge, Katia Hazim N. Abu Shihab, Basil H. Aboul-Enein, Nada Benajiba, MoezAlIslam Faris

**Affiliations:** 1 College of Professional Studies, University of New England, Applied Nutrition Graduate Program, Maine, USA; 2 Department of Clinical Nutrition and Dietetics, College of Health Sciences, University of Sharjah, United Arab Emirates; 3 College of Arts & Sciences, University of Massachusetts Dartmouth, Health & Society Program, Massachusetts, USA; 4 London School of Hygiene & Tropical Medicine, Faculty of Public Health and Policy, UK; 5 Ibn Tofail University-CNESTEN, Joint Research Unit in Nutrition and Food, RDC-Nutrition AFRA/IAEA, Kenitra, Morocco

**Keywords:** university students, Arab, nutrition interventions, Middle East, North Africa

## Abstract

The university setting is an ideal setting to implement nutrition education interventions. Because the transition to college life often overlaps with young adults’ increase in independence in food choice and nutrition related behavior, this is an optimal time to target this population and several models exist to best utilize the college setting to support nutrition education interventions. This review aimed to examine the effectiveness of nutrition interventions in university student populations across the League of Arab States. A scoping review following the PRISMA-ScR guidelines was conducted across 14 databases for relevant publications published through May 2023 to find studies conducted across Arab countries. Quality assessment was conducted using the Effective Public Health Practice Project tool. Seven primary studies were included in this review. Although the interventions and outcome measures were disparate, there were positive impacts of the interventions, including improved nutrition knowledge and nutrition habits, knowledge of nutrient sources, and knowledge about the relationship between diet and disease. Recommendations to maximize the efficacy of nutrition interventions conducted across the League of Arab States include purposeful design of the intervention based on a theoretical framework, the use of a control group in the research design, and follow-up measures to examine post-intervention effects. Consideration should also be given to intervention duration and availability in college-students’ schedules. Adaptation of intervention materials, measures and delivery methodologies should emphasize local foods, food culture and dietary practices.

## Introduction

Nutrition status is necessary for good health, and nutrition behaviors, many developed in childhood and adolescence, may predict obesity and associated comorbidities, such as Type II diabetes, cardiovascular disease, hypertension and increased risk of some cancers, as we age (1–3). The United Nations Sustainable Development Goals are 17 goals designed to foster prosperity, equity and sustainability against a number of health and sustainability domains (4); two goals, ‘no hunger’ and ‘good health and well-being’, are amongst the most directly related to nutrition. Good health metrics include the management and prevention of obesity and overweight, which have been linked to several chronic health issues, particularly later in life (4). Several developing countries that previously faced a high rate of undernutrition are currently double burdened with undernutrition and overnutrition, both of which pose public health risks for malnutrition among their respective populations (5–7).

The League of Arab States has experienced industrialization, dietary transition and a modernized lifestyle that includes physical inactivity and increased consumption of convenience foods which have contributed to the increased obesity rates in this region (8,9). In oil-producing countries of the Arab Gulf (Kuwait, Qatar, United Arab Emirates and Saudi Arabia), obesity among adult men hovers around 30% with higher rates (40%) among adult women (10). Published reviews estimated the 2013 prevalence of overweight and obesity in the region in boys and girls <20 years old to be 22.2% and 27.9%, respectively (11,12).

The university setting is an ideal setting in which to implement nutrition education interventions that may impact nutrition knowledge and behavior. Nutrition behavior is complex and is defined by Hummel and Hoffmann (13:p.1) as ‘the sum of all planned, spontaneous, or habitual actions of individuals or social groups to procure, prepare, and consume food as well as those actions related to storage and clearance. In this context, the term “nutritional behavior” not only refers to influencing factors but also to health, environmental, social, and economic implications along the entire product chain from farmer to consumer’ and thus, for the purposes of the studies included in this review, comprises any nutrition or diet related behavior explicated by the studies’ researchers as a nutrition-related behavior of investigatory interest.

A systematic review conducted across eight countries in 2015 with university and college students found that half of the nutrition interventions included established improvement in nutrition behavior, although due to heterogeneity and a lack of standard methodology, a meta-analysis of the findings could not be completed (14). Another 2018 systematic review (15), assessing US-based nutrition interventions in college students, concluded that nutrition behavior change could be positively impacted in the university setting, and those that were most effective elucidated the theoretical framework used and incorporated a control group into the research design (15). Plotnikoff *et al*. (14) noted that interventions of a duration <12 weeks seemed to be most acceptable to university students, Brace *et al.* (15) suggested that follow-up to investigate effect after the intervention has ended is critical to understanding whether the behavior change persisted post-intervention. Intervention duration may be a critical component in this population, as students may be busy with school, work and social obligations. A 2018 feasibility study of a one-session web-based intervention based on the PRECEDE–PROCEED framework found both high feasibility and acceptability in Australian college students; there was a significant decrease in the intervention group percentage/day consumption of discretionary foods (*p* = 0.012) compared with the control, indicating that even short interventions have the possibility to effect change (16). Another web-based nutrition intervention offered in 2005 over 1.5–2 h to US college students (depending on the experimental group) found significant improvements in fruit and vegetable intake in the experimental groups and significant advancement in stages of change as related to nutrition behavior (17). However, interventions designed to be offered parallel to a college student’s schedule may also provide opportunities to effect nutrition behavior change. Using a class-based approach and the Social Cognitive Theory, US college students that participated in a sophomore-level 15-week basic nutrition course with an emphasis on healthful dietary choices and disease prevention significantly increased their fruit and vegetable consumption (18). Using a similar model, a 2009 course-based intervention over the duration of a semester significantly improved healthful eating and vegetable intake in US college students while decreasing high-fat dairy consumption (19). Another study, comparing the effects of a semester-long health education course offered in-person or online, found significant increases in fruit and vegetable intake, bran, whole grain and brown rice intake, with stronger improvements in the in-person course (20). Because the transition to college life often overlaps with young adults’ increase in independence in food choice and nutrition related behavior, this is an optimal time to target behaviors in this population and several models exist to best utilize this setting to support nutrition education interventions. While several reviews have been conducted examining nutrition interventions in the college setting and among university students in the US and other parts of the world (14,15,21–24), to our knowledge, no review to date has been conducted in the university setting across Arabic-speaking countries. This review fills that gap.

## Methods

### Selection criteria

Population, Intervention, Comparison, Outcomes, and Study (PICOS) formatting was utilized to develop the inclusion and exclusion criteria for this review (see Table 1). The search was conducted in the spring of 2023 and the results communicate literature published through May 2023.

**Table 1.** PICOS criteria for inclusion and exclusion of studies.

Parameter	Inclusion criteria	Exclusion criteria
**Date range**	Up to and including 31 May 2023	N/A
**Population**	University/college-aged students who were examined in any country of the Arab League	Non-university/college students Arab students who are not studying in an Arab League country Students undergoing medical nutrition therapy-based diets
**Intervention type**	Any university-based intervention that addresses nutrition-related aspects	Interventions that are not based on college facilities Interventions that do not address nutrition-related outcomes
**Comparators**	Pre-intervention, baseline nutrition-related variables (i.e. anthropometric measures, biochemical parameters, nutrition-related knowledge, dietary habits, perceived hunger)	Controlled, received no intervention, or received partial intervention (e.g. compared two levels of intervention)
**Outcomes of interest**	Changes in anthropometric outcomes. e.g. BMI for age, height for age Changes in nutrition-related knowledge Changes in adhering to dietary macronutrient and/or micronutrient recommendations Changes in adherence to healthy dietary habits and avoidance of unhealthy ones	Non-nutrition knowledge or weight related outcomes
**Language**	English, Arabic or French	All other languages
**Study type**	Experimental intervention studies with quantitative outcomes Peer-reviewed original research articles Original research conference publications	Non-peer-reviewed articles Non-numeric/categorical assessments or qualitative studies Commentaries Narratives Communications Non-intervention based studies White papers Similar article types Grey literature

PICOS: Population, Intervention, Comparison, Outcomes, and Study; BMI: body mass index; N/A: not applicable.

### Search procedures

For this systematic scoping review, we used Preferred Reporting Items for Systematic Reviews and Meta-Analyses for Scoping Reviews guidelines (25) and began with a comprehensive search within 14 biomedical bibliographic databases (see Table 2) using combination strategies of subject heading keywords, terms, phrases, and Boolean operators (Table 2). Search strategies using the selected databases (see Table 2) were adapted according to the indexing systems of each respective database. For the purpose of this review, Arabic-speaking countries were defined as the 22-member countries of the League of Arab States (26).

**Table 2.** Electronic databases used with relevant search period and terms.

Databases	Search period	MeSH keywords, terms, phrases, and Boolean operators^a^
EBSCOHost; BIOSIS; CINAHL; ScienceDirect; ArticleFirst; Biomed Central; BioOne; ProQuest; SAGE Reference Online; Scopus; SpringerLink; PubMed; Taylor & Francis; Wiley Online	Up to 31 May 2023	**(ALL FIELDS)** “university”; “university-based”; “college”; college-based”; “students” AND **(ALL FIELDS)** “nutrition”; OR “diet” AND **(ALL FIELDS)** “education” OR “educat” OR “promotion” OR “promot” OR “intervention” OR “program” AND **(ALL FIELDS)** “Algeria” OR “Egypt” OR “Bahrain” OR “Comoros” OR “Djibouti” OR “Iraq” OR “Jordan” OR “Saudi Arabia” OR “Kuwait” OR Lebanon” OR “Libya” OR “Mauritania” OR “Morocco” OR “Oman” OR “Palestinian Territories” OR “Qatar”; “Yemen” OR “Somalia” OR “Sudan” OR “Syria” OR “Tunisia” OR “United Arab Emirates”

a Based on the Preferred Reporting Items for Systematic Reviews and Meta-Analyses for Scoping Reviews, the same search strategy was employed in each of the 14 databases listed using all of the keywords, search terms, and phrases included above. Languages searched include English, Arabic, or French.

### Quality assessment

**Figure 1.**
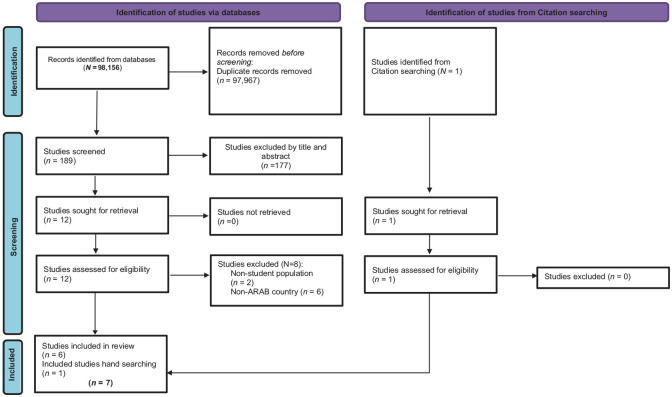
PRISMA-ScR flow diagram. PRISMA-ScR: Preferred Reporting Items for Systematic Reviews and Meta-Analyses for Scoping Reviews

Two of the authors conducted the searches for relevant studies and one author utilized Rayyan QCRI software (27) to assist in the screening process. All retrieved articles were screened for relevance to the topic. In addition, reference lists from retrieved studies were also hand reviewed to identify any additional relevant publications (see Figure 1). Titles and abstracts were screened for relevancy, and potentially relevant journal abstracts were reviewed by three of the authors. Potential articles for inclusion in this review were evaluated independently for relevance, merit and inclusion/exclusion criteria. Studies accepted for inclusion were individually reviewed by each author. Additionally, each study was assessed by an independent reviewer. Any disagreement was resolved by consensus or by a second reviewer. Quality assessment was conducted using the Effective Public Health Practice Project (EPHPP) tool, which allows authors to evaluate the study quality on the metrics of study design, analysis, withdrawals and dropouts, data collection practices, selection bias, invention integrity, blinding as part of a controlled trial, and confounders (28).

### Methodological quality

The quality of the seven intervention studies was assessed using the EPHPP tool (28) and it was found that one study (29) was of strong quality, five studies (30–34) were moderate in overall quality and one (35) was weak in overall quality. All of the studies failed to explicitly meet a ‘strong’ rating in at least one of the criteria measured by EPHPP. All of the studies scored weak in selection bias, given their convenience sampling to recruit participants. A pre–post intervention design is used in the majority of the studies (*n* = 6) (30–35). One study (29) utilized a non-randomized feasibility study with two arms. Most of the studies (29,30,32–35) (*n* = 6) scored strongly on the data collection tools criterion, and one (31) scored as moderate as it did not address the validity of the 24-h food recall protocol used.

## Results

### Characteristics of the studies

Seven primary research manuscripts were included in this scoping review. Four studies were conducted in the Gulf Cooperation Council with two studies (31,34) from Saudi Arabia, and two studies (29,32) from the United Arab Emirates. Also included were one study from Jordan (30) and two studies (33,35) from Lebanon. The summary findings and intervention setting are tabulated in Table 3. The sample sizes of the analyzed studies ranged from 16 participants to 3555 participants. Only one study (29) evaluated its intervention process (i.e. implementation, encountered barriers, participants’ opinions and appreciation, and overall feasibility of the intervention), as well as the results of the intervention.

**Table 3.** Study design, overall study quality and overall intervention effectiveness on anthropometrics and dietary behavior included primary interventional studies (*N* = 7).

First author (reference)/country	Study design/*N*	Overall study quality	Overall effectiveness on anthropometrics^a^	Overall effectiveness on dietary knowledge and/or behavior^a^	Overall effectiveness on other assessed health attitudes, behaviors and effects^a^
Educational					
Soweid *et al*. (35)/Lebanon	Quasi-experimental pre–post-test/*N* = 16^b^	Weak	N/A	Nutritional knowledge, behavior and attitude improved by 13.1% from pre-intervention to post-intervention. (No significance test executed^b^.) Six out of 14 students moved at least one stage forward and five students stayed in the maintenance stage on the trans-theoretical model of change regarding fruits and vegetable intake (++)	Over 20% change from pre-intervention to post-intervention in knowledge, behavior and attitude: stress management, sexual health, health care consumerism, environmental health (++). (No significance test executed^b^.) Six out of 15 students moved one level further in the trans-theoretical model of change in terms of exercise
Abu-Moghli *et al*. (30)/Jordan	Quasi-experimental pre–post-test/*N* = 130	Moderate	Students with lower BMI had higher health practice scores (*p* = 0.02)	Nutrition habits significantly (*p* = 0.00) improved in the experimental group (++) compared with the control group.	No significant difference found in physical activity from pre–post-test between the experimental and control groups (0)
Ali *et al*. (29)/UAE	Non-randomized feasibility study with two arms/*N* = 161	Strong	Significant reduction in waist circumference (*p* < 0.001), BMI (*p* < 0.042) and body fat (*p* < 0.001) in the enhanced intervention group (++). Fat-free mass significantly increased in enhanced intervention group (++)	Dietary knowledge of nutrients’ sources (*p* < 0.001) significantly increased in the enhanced group (++). Self-efficacy in reducing sugar significantly (*p* = 0.005) increased in the enhanced intervention group (++).	In the enhanced intervention group, physical exercise was significantly (*p* = 0.025) increased, while sitting time was significantly (*p* < 0.001) decreased (++). Self-efficacy to overcome barriers to physical exercise was significantly (*p* = 0.012) increased (++)
Ismail *et al*. (32)/UAE	Experimental one-group, pre–post-test/*N* = 90	Moderate	N/A	Significantly improved knowledge and practices related to salt intake (purchasing of low salt foods (*p* = 0.022), rarely adding salt to food during cooking (*p* = 0.019) and at the table (*p* = 0.011), trying to reduce salt intake (*p* = 0.014) and trying to use spices in place of salt (*p* = 0.044) all increased) (++). Behavior was still significant, but demonstrated declines at one-month follow-up	N/A
Maddah *et al*. (33)/Lebanon	Quasi-experimental pre–post-test/*N* = 156	Moderate	No change in BMI in intervention compared with control group (0)	Intake of processed foods significantly decreased (*p* = 0.03) in the intervention group (++)	No difference in high-calorie intake or hot beverage consumption in the experimental compared with the control group (0)
Mumena *et al.* (31)/Saudi Arabia	Quasi-experimental pre-post-test/*N* = 46	Moderate	N/A	The intervention group consumed significantly (*p* = 0.001) less sugar (++) and the sugar contribution to total energy intake was significantly (*p* = 0.001) less in the intervention group (++)	N/A
Educational and environmental					
Alzaben *et al*. (34)/Saudi Arabia	Pre–post-test, non-randomized experimental study design/*N* = 3555	Moderate	No change in weight in the intervention group (0).	The mean score for students was significantly (*p* = 0.016) higher in nutrition knowledge after intervention (++), with no significant change in nutrition practice (0).	N/A

(++) = positive desirable change; (–) = negative undesirable change; (0) = no effect.

a All changes written refer to the reported significant results unless otherwise stated.

b Sample too small to report statistical significance.

BMI: body mass index; N/A: not applicable or not assessed; UAE: United Arab Emirates.

### Intervention characteristics

Two studies’ interventions (29,35) were theory-based, while four (30,31,33,34) allude to using the acquisition of knowledge to predict attitudes and behaviors as the basis for the pre–post measures and one (32) explicitly states using Knowledge, Attitudes and Practices. Of the theory-based interventions, one (35) used the framework of the trans-theoretical model of change and the other (29) social-cognitive theory. As a part of the assessment, Soweid *et al*. (35) had participants (*N* = 16) report the stage they were at based on the trans-theoretical model of change regarding fruit and vegetable intake, regular exercise and tobacco use. Meanwhile, Ali *et al*. (29) modeled their web-based/app-based intervention program upon the social-cognitive theory framework, targeting constructs such as self-monitoring, self-efficacy and social support.

Most studies (29,31,35) implemented and evaluated solely educational interventions, offered in the form of educational sessions, distribution of educational materials and/or through educational curriculum. While no studies implemented purely environmental interventions, that is, changes to the university environment, one study (34) did add an environmental component to the intervention, using motivational messages on television screens around the university campus. This study was the only study to change the university’s nutrition policy by requesting the university food vendors to change the sold food to healthier alternatives, avoid false food labelling and adopt healthier cooking methods.

Overall, the included studies’ intervention programs were conducted via educational on-campus sessions, with only one (29) delivering its educational program online. The studies’ intervention durations ranged from two five-day training programs to 16 months, with no follow-up assessment being indicated in any of the studies. In several of the studies (29–34), the research team conducted the intervention and education. Only one (29) involved experts, specifically nutritionists, in intervention delivery. Soweid *et al*. (35) evaluated an intervention delivered by the course instructor, evaluating a health awareness course’s impact on dietary and health-related behavior and knowledge of students. Five of the included studies (29,30,33–35) addressed several nutrition and diet related outcomes in their intervention programs, while one study (31) specifically addressed excessive intake of added sugars and another investigated reduction of sodium intake (32).

### Evidence of effect

The components that were used to assess the effect for the reviewed studies are shown in Table 3. Given that there were only seven studies (29–35), and that the research design and outcome measures varied so widely, evidence of the effect of nutrition interventions in university based settings across the League of Arab States is determined to be inconclusive. The timing of each intervention varied from two five-day training programs to 16 months; however, intervention timing did not appear to impact the intervention findings. Most of the studies (29,30,33,34) assessed both anthropometric and dietary-related changes, while other studies (29,31,32,35) assessed nutrition-related changes in nutrition practices/behaviors, nutrition knowledge or both. The tools used to assess post-intervention dietary behavior or knowledge were all self-report surveys and questionnaires; two studies (29,30) used a general nutritional knowledge questionnaire, one (31) used 24-h dietary recall, one (35) used a Comprehensive Health Assessment Inventory and one study (34) adapted a previous nationwide survey utilized by the Saudi Food and Drug Authority.

Nutrition knowledge increased in four (57%) of the studies, with Ali *et al*. (29), Ismail *et al*. (32), Mumena *et al*. (31) and Alzaben *et al*. (34) reporting significant improvements in knowledge related to nutrient sources (*p* < 0.001), salt intake (purchasing of low salt foods (*p* = 0.022), rarely adding salt to food during cooking (*p* = 0.019) and at the table (*p* = 0.011), trying to reduce salt intake (*p* = 0.014) and trying to use spices in place of salt (*p* = 0.044)), sugar consumption (*p* = 0.001) and general nutrition knowledge (*p* = 0.016), respectively (16–19). Nutrition behavior/habits were positively changed in two (28.6%) of the studies with Abu-Moghli *et al*. (30) reporting that there was significant improvement in dietary habits in the experimental (*p* < 0.00) compared with the control group and Maddah *et al*. (33) noting a significant decrease in processed food intake in the intervention (*p* = 0.03) compared with the control group. The study by Soweid *et al*. (35) was too small to meaningfully report on statistical significance but did determine that the post-intervention findings support the potential positive impact of a health awareness class on nutrition knowledge and intent to eat fruits and vegetables. Although the interventions and outcome measures were disparate, there were positive impacts of the interventions, including improved nutrition knowledge (34) and nutrition habits, (30) significant decreases in waist circumference along with significant increases in knowledge of nutrient sources, knowledge of the relationship between diet and disease, and an increase in social support to decrease fat intake (29), and a significant decrease in sugar consumption (31). These findings, taken together, show an inconclusive yet positive trend for university-based nutrition education interventions to impact nutrition and health behavior.

## Discussion

While several systematic reviews address nutrition interventions in the university setting, including in countries such as the USA (15,21,22,36), Turkey, Jordan, Lebanon, Scotland, Ireland, Taiwan and Australia (14), Peru, the UK (21), Israel and Korea (36), to the authors’ knowledge, this is the first review to examine the effects of nutrition interventions with this population specifically, offered across the League of Arab States. It has been found that university and college settings may offer nutrition educators an ideal target population and location in which to conduct nutrition education interventions (14,15). While an article by Pulimeno *et al*. (37) focuses on what makes K-12 school settings ideal for health promotion education, including nutrition interventions, some of the specific reasons for this (peer and community support opportunities, ability to advocate for environmental change to support health and well-being, motivation at pivotal moments of growth and development of life-long learning skills) apply to university and college settings as well. There is also an opportunity to reach a diverse population in terms of race, ethnicity, gender, socioeconomic and cultural backgrounds (38). The inclusion of nutrition and health promotion coursework in the university setting has shown promise in increasing food security (39), nutrition knowledge and counseling skills (40) and healthful eating behaviors (18,41) in college students. While eating disorders and disordered eating are found to be prevalent in university-level nutrition and dietetics students (42), it has also been found that as they proceeded through their program’s coursework, they tend to adopt healthier eating behaviors, while obsessive food tendencies decrease (43). Further, college and university-based interventions delivered outside of a traditional course work setting have also been shown to improve healthful eating in college student populations (14,15,36,44). Because university students are transitioning into adulthood and becoming more autonomous in food choice and behavior, even basic environmental interventions such as point of purchase interventions (22), color-coded vending machine options (44) and promotional marketing, messaging and price interventions (45) can positively impact food choice and nutrition behavior. Based on the findings of others, these short duration (14,46) and/or environmental initiatives (45) have the ability to positively impact nutrition behavior and food choice in university and college students. Using the literature from successful nutrition education interventions across universities and colleges can help inform the development and cultural tailoring of nutrition education interventions to be accessible, socially acceptable and successful when implemented in the League of Arab States.

## Strengths and limitations

The League of Arab States have experienced industrialization, nutritional transition and a modernized lifestyle which have contributed to the increased obesity rates, with countries of the Arab Gulf (Kuwait, Qatar and Saudi Arabia) demonstrating high rates of obesity among adult men (30%) and adult women (40%) (10). Nutrition education interventions may provide a way to promote knowledge and health through improving nutrition knowledge, dietary behaviors and habits. While several reviews have examined nutrition interventions in the college setting in the USA and other parts of the world (14,15,21–24), no review to date has been conducted in the university setting across Arabic-speaking countries. This review fills that gap. Strengths of this review include the number of databases used, the three languages used in the search and that the article quality was assessed using the EPHPP. Limitations include the limited number of articles that met inclusion criteria, and heterogeneity in the approach to intervention design, intervention duration and outcomes measured. One article had a small sample size, precluding analysis for statistical significance of the findings.

## Conclusion

This scoping review found limited studies evaluating the effectiveness of nutrition interventions targeting university-level student populations across the League of Arab States. These findings show an inconclusive yet positive trend for university-based nutrition education interventions implemented in the League of Arab States to impact nutrition and health behavior. However, successful nutrition interventions outside the League of Arab States have been conducted in the university setting and may be adaptable to serve this specific population. There are specific recommendations to maximize the efficacy of nutrition interventions to positively impact nutrition knowledge, practices and behaviors, and anthropometrics such as weight, waist circumference, waist to hip ratio and body mass index; these include purposeful design of the intervention to include a theoretical framework, the use of a control group in the research design, and follow-up measures to examine post-intervention effects (14,15). Consideration should also be given to duration and availability of college-students’ schedules, with some successful interventions being short in duration, offered online, or environmental (16,17,20,44–46). The authors propose that future investigations into the efficacy of university-based nutrition interventions offered to university-level participants in the League of the Arab States apply these recommendations. Further, adapting intervention materials, measures and delivery methodologies to address local foods, food culture and dietary practices would likely impact the acceptability and applicability to the target audience, thus potentially impacting knowledge acquisition, adoption of healthful nutrition related practices and behaviors, and the longevity of the impact of the intervention.
